# Simplification or simulation: some unclear issues in sample size calculation

**DOI:** 10.1186/1745-6215-14-S1-O100

**Published:** 2013-11-29

**Authors:** Chao Huang

**Affiliations:** 1 Cardiff University, Cardiff, UK

## 

In the clinical trial designing stage, trial statisticians need to provide a reference sample size for conducting the trial. In general, this task could be completed by forming the main research question into a statistical procedure and then implementing the published formulae or software, such as n-query and STATA, to make the calculation. When more complex statistical procedures are involved in the trial, the existing formulae or software may become not available any more. Some statisticians fill this gap by assuming an alternative simpler statistical procedure, while other statisticians conduct some simulations to generate sample size estimates. However, it is still unclear which approach is more recommendable in practice. To this end, we conduct a study to address three corresponding issues in sample size calculation. The first one is whether the software and/or standard formulae always give correct answer, or the simulation results are more reliable. The second one is to assess the reliability of calculating sample size by assuming a simpler statistical procedure. The last one is, in the simulation cases, whether we should fix the values of some components, or randomize them in a feasible region. Some recommendations and guidance are provided and shared to other researchers.

